# Imaging of C-fos Activity in Neurons of the Mouse Parietal Association Cortex during Acquisition and Retrieval of Associative Fear Memory

**DOI:** 10.3390/ijms22158244

**Published:** 2021-07-31

**Authors:** Olga I. Ivashkina, Anna M. Gruzdeva, Marina A. Roshchina, Ksenia A. Toropova, Konstantin V. Anokhin

**Affiliations:** 1 Institute for Advanced Brain Studies, Lomonosov Moscow State University, 119991 Moscow, Russia; xen.alexander@gmail.com (K.A.T.); k.anokhin@gmail.com (K.V.A.); 2 National Research Center “Kurchatov Institute”, 123182 Moscow, Russia; annadronova@mail.ru; 3 Laboratory for Neurobiology of Memory, P.K. Anokhin Institute of Normal Physiology, 125315 Moscow, Russia; 4 Institute of Higher Nervous Activity and Neurophysiology of RAS, 117485 Moscow, Russia; marina.zots@gmail.com

**Keywords:** parietal association cortex, fear memory, c-fos, calcium activity, transgenic mice, two-photon imaging

## Abstract

The parietal cortex of rodents participates in sensory and spatial processing, movement planning, and decision-making, but much less is known about its role in associative learning and memory formation. The present study aims to examine the involvement of the parietal association cortex (PtA) in associative fear memory acquisition and retrieval in mice. Using ex vivo c-Fos immunohistochemical mapping and in vivo Fos-EGFP two-photon imaging, we show that PtA neurons were specifically activated both during acquisition and retrieval of cued fear memory. Fos immunohistochemistry revealed specific activation of the PtA neurons during retrieval of the 1-day-old fear memory. In vivo two-photon Fos-EGFP imaging confirmed this result and in addition detected specific c-Fos responses of the PtA neurons during acquisition of cued fear memory. To allow a more detailed study of the long-term activity of such PtA engram neurons, we generated a Fos-Cre-GCaMP transgenic mouse line that employs the Targeted Recombination in Active Populations (TRAP) technique to detect calcium events specifically in cells that were Fos-active during conditioning. We show that gradual accumulation of GCaMP3 in the PtA neurons of Fos-Cre-GCaMP mice peaks at the 4th day after fear learning. We also describe calcium transients in the cell bodies and dendrites of the TRAPed neurons. This provides a proof-of-principle for TRAP-based calcium imaging of PtA functions during memory processes as well as in experimental models of fear- and anxiety-related psychiatric disorders and their specific therapies.

## 1. Introduction

The parietal cortex is an associative cortical area that participates in various integrative brain functions, including multisensory processing, decision-making, motion planning, navigation, attention, and working memory [1]. Though this area has been extensively studied in cognitive tasks in primates [2], only more recently has it become the subject of corresponding analyses in rodents [3,4].

The parietal region in the rodent brain is defined as an area between visual and somatosensory cortices [3,5,6]. It is connected with diverse brain areas, including other associative cortical regions, such as orbitofrontal, retrosplenial and anterior cingulate cortices [4,7]. Its neurons respond to modality-specific (auditory, visual, or somatosensory) as well as complex stimuli [8,9,10]. In rodents, this area is also involved in spatial navigation [11] and decision-making [12,13].

According to Franklin and Paxinos (2007), the mouse parietal cortex consists of anterior and posterior parts, and the anterior part corresponds to the parietal association cortex (PtA). Though most rodent studies address the functions of the posterior parietal cortex, network analysis of mouse cortical connectivity revealed that the PtA has a high level of betweenness centrality that makes it a strong hub region within the cortical network [14]. Therefore, in this study, we focus on the role of mouse PtA in associative memory processes.

Retrosplenial, cingulate, and frontal associative cortical areas, which send projections to the PtA, are involved in the coding and retrieval of different types of memory [15,16,17,18,19]. Interestingly, there is no evidence about direct connections between the PtA and the prelimbic prefrontal cortex, which is known to be involved in the formation and retrieval of associative memory including conditioned fear memory in rats in mice [20,21,22]. The rat PtA is known to participate in the retrieval of recent and remote spatial memory [23,24]. However, the contribution of PtA to associative memory is still poorly understood. A direct way to address this question is to examine the specific expression of immediate-early genes (IEGs) involved in experience-dependent neuronal plasticity.

Expression of IEGs such as *c-fos* is commonly used to identify neurons activated by learning and involved in memory encoding [25,26,27]. *c-fos* encodes the transcription factor that regulates the activity of effector genes and the following long-term plasticity in neurons [28]. Different Fos-based methods are used to investigate the experience-induced changes of brain neuronal circuits in various learning and memory paradigms. C-Fos immunostaining is commonly used to access neuronal activity only at a single time point. In contrast, in vivo Fos-imaging allows observing the activation of the same neuronal population in different behavioral episodes. In this case, Fos-EGFP transgenic mice allow repeated imaging of Fos-positive neurons and comparison of neuronal populations activated during learning and memory retrieval [18,29,30,31]. Similarly, the method of targeted recombination in active populations (TRAP) was used to capture the candidate engram neurons [25,32]. TRAP is an approach to obtain permanent genetic access to distributed neuronal ensembles that are activated by experiences within a limited time window [32,33]. In Fos-TRAP transgenic mice, the tamoxifen-dependent recombinase CreERT2 is expressed in an activity-dependent manner under the control of the *c-fos* promoter. Active cells that express CreERT2 undergo recombination only in the presence of tamoxifen (TM). This allows genetic access to neurons that were active during a time window less than 24 h after injection. Nonactive cells do not express CreERT2 and do not undergo recombination, even if TM was injected [32]. Previously the TRAP approach was used to assess the involvement of neurons genetically captured during context fear conditioning (FC) in subsequent memory retrieval [33,34]. Optogenetic silencing of TRAP-labeled neuronal populations in CA3 or DG prevented the expression of the corresponding memory [33]. In the present study, we used c-Fos immunostaining, Fos-EGFP in vivo imaging, and Fos-TRAP to investigate the involvement of PtA cortex in the encoding and retrieval of associative fear memory in mice. Using c-Fos-immunohistochemistry, we found that PtA neurons were specifically activated during cued fear memory retrieval. In vivo Fos-EGFP imaging showed, in addition, the specific changes of activity during both fear conditioning and memory retrieval. Finally, we applied the TRAP technique for calcium imaging specifically in PtA engram cells. We showed no specific activity in such cells during conditioned stimulus (CS) presentation during the 1-day-old memory retrieval.

## 2. Results

### 2.1. Immunohistochemical Analysis of Fos Expression in the PtA

**Figure 1:**
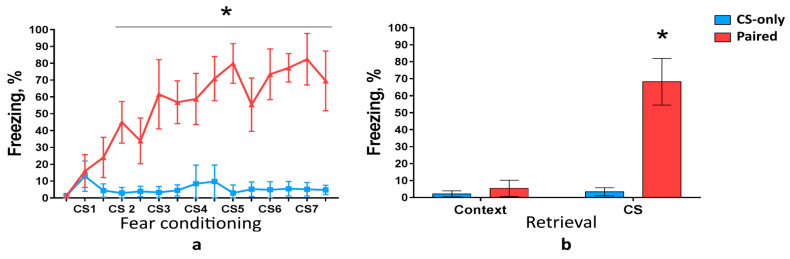
Freezing during (**a**) fear conditioning (FC) and (**b**) memory retrieval 24 h after the FC (mean, 95% confidence interval (CI)). Fear response increased during training only in the Paired group (* *p* < 0.0001, two-way ANOVA). Trained animals froze more than CS-only group during conditioned stimulus (CS) memory retrieval (* *p* < 0.0001, two-way ANOVA with post hoc Tukey test).

First, we analyzed c-Fos expression in the PtA during cued fear memory acquisition and retrieval. Wild-type mice were trained to associate CSs with foot shocks. We used three groups of mice: Paired (CS was paired with foot shock), CS-only (mice received only CS), and Home Cage (HC, mice were not tested in FC assay). Only Paired group but not CS-only group developed freezing behavior as the number of pairings increased (two-way ANOVA, *p* < 0.0001) (Figure 1a). Freezing response to the CS during memory retrieval was higher in the Paired group compared with CS-only group (two-way ANOVA, *p* < 0.0001) (Figure 1b). Freezing level during novel context exploration, however, was the same in the Paired and CS-only groups (two-way ANOVA, *p* = 0.5341), (Figure 1b). These results demonstrate that mice learned association between conditioned and unconditioned stimuli (CS-US association) and did not exhibit memory generalization to a novel context.

**Figure 2:**
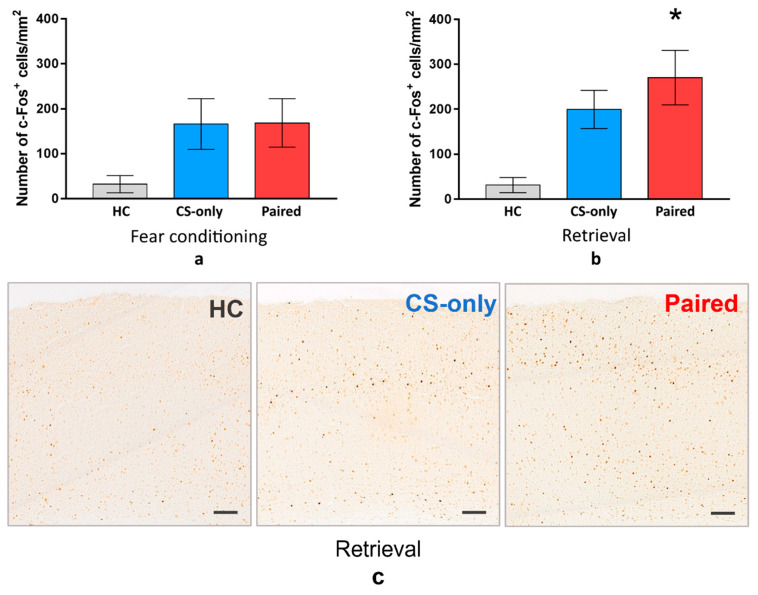
The density of c-Fos-positive cells in the PtA 1 h after (**a**) fear learning and (**b**) memory retrieval on the next day (mean, 95% CI). The density of the PtA c-Fos-positive cells was higher in the Paired and CS-only groups than in the Home cage (HC) group during FC or memory retrieval (*p* < 0.001, one-way ANOVA and post hoc Tukey test). During memory retrieval, c-Fos-positive cells density was higher in the Paired group compared with the CS-only group (* *p* = 0.0364, one-way ANOVA and post hoc Tukey test). (**c**) Image of Fos-positive cells in PtA in HC, CS-only and Paired groups (scale bar is 100 μm).

For the next step, we sacrificed the animals from all groups one hour after FC or memory retrieval and performed immunostaining to detect c-Fos in the PtA. The density of the PtA c-Fos-positive cells was higher in the Paired and CS-only groups than in the HC group during FC or memory retrieval (one-way ANOVA and post hoc Tukey test, *p* < 0.001) (Figure 2). However, there was no significant difference between Paired and CS-only groups during FC (one-way ANOVA and post hoc Tukey test, *p* = 0.9532) (Figure 2a). On the contrary, c-Fos-positive cell density was higher in the Paired group than in the CS-only group during memory retrieval (one-way ANOVA and post hoc Tukey test, *p* = 0.0364) (Figure 2b). This data suggest that PtA neurons are active during new experience acquisition regardless of the associative or nonassociative nature of this experience. At the same time, PtA is specifically activated during CS memory retrieval, suggesting its role in the recent fear memory storage.

### 2.2. In Vivo Investigation of C-Fos Expression in the PtA of Fos-EGFP Mice

**Figure 3:**
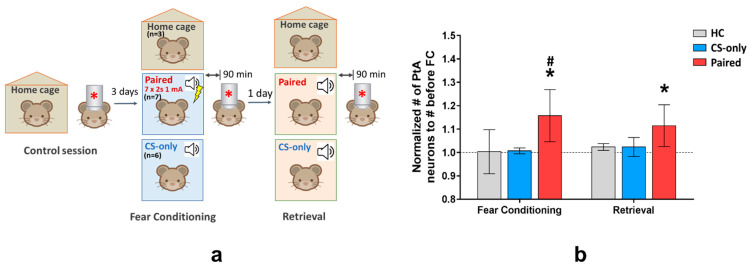
(**a**) Design and timeline of the in vivo imaging experiment with Fos-EGFP mice. (**b**) Number of PtA Fos-EGFP positive neurons normalized to the number of neurons active during the control session (mean, 95% CI). The number of Fos-EGFP positive neurons was increased during learning and memory retrieval on the next day in the Paired group (*n* = 7) but not in the control groups (* *p* = 0.0156, compared with 1, *t*-test; # *p* < 0.05 compared with CS-only (*n* = 3) and HC group (*n* = 3) groups, two-way ANOVA and post hoc Tukey test).

In the next experiment, we analyzed c-Fos activation of individual PtA neurons during cued memory acquisition and retrieval using in vivo two-photon imaging in Fos-EGFP transgenic mice. Imaging was performed three days before FC to access basal level of c-Fos expression in PtA neurons in a home cage, 90 min after FC session, and 90 min after memory test (Figure 3a). This approach allowed us to address and compare c-Fos activity of a specific neuronal population in PtA after different behavioral procedures in the same animal.

Overall, we identified 9325 neurons in all the mice during all sessions of two-photon visualization. In the control imaging session (home cage condition, 3 days before fear conditioning) 520 ± 160 Fos-EGFP positive neurons per mouse (mean ± 95% CI) in 16 mice were identified. 17% of all identified neurons changed their activity (i.e., were activated or inactivated) at least in one imaging session. We normalized the number of Fos-EGFP positive neurons in both conditioning and retrieval sessions to the number of neurons that were active during the control imaging session and found that the number of Fos-EGFP positive neurons increased during the FC training and memory recall in the Paired group but not in the CS-only and HC groups (*t*-test, compared with 1, *p* = 0.0156) (Figure 3b). Moreover, we found that during learning, but not during memory retrieval, the number of Fos-EGFP positive PtA cells was significantly higher in the Paired group than in the CS-only or HC groups (two-way ANOVA and post hoc Tukey test, *p* < 0.05).

**Figure 4:**
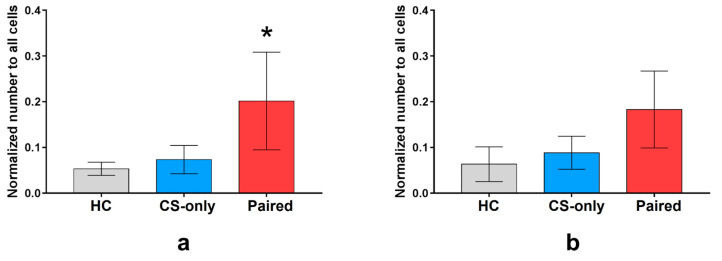
Number of the PtA neurons that were inactive before FC and active during (**a**) fear learning and (**b**) memory retrieval on the next day normalized to all identified neurons in Fos-EGFP mice (mean, 95% CI). The number of neurons that were inactive before FC and were active during FC was higher in the Paired group (*n* = 7) than in CS-only (*n* = 6) and HC (*n* = 3) groups (* *p* < 0.05, one-way ANOVA and post hoc Tukey test).

Next, we compared the number of neurons that were inactive during the control imaging session, but were activated during FC or memory test. The number of neurons activated during fear conditioning normalized to the total number of neurons for each group was higher in the Paired group than in the CS-only and HC groups (64 (*n* = 7 mice), 40 (*n* = 6), and 32 (*n* = 3) neurons in average, respectively, one-way ANOVA and post hoc Tukey test, *p* < 0.05) (Figure 4a). The number of neurons activated by memory retrieval was similar in all groups (64 (*n* = 7), 49 (*n* = 6), and 38 (*n* = 3 mice) neurons on average, respectively, one-way ANOVA, *p* = 0.2185) (Figure 4b).

Altogether, these results indicate that, as sampled by in vivo Fos-EGFP activity, PtA neurons are involved in associative fear memory formation and retrieval in the mouse brain.

However, there are certain restrictions with the use of c-Fos as an indicator of conditioned neuronal activity. Long duration of c-Fos protein synthesis or of Fos-triggered EGFP accumulation does not allow precise matching of these biochemical responses to the stimuli that induced them. Furthermore, not all neuronal responses are accompanied by induction of *c-fos* transcription. To overcome these limitations and to address the causal links between learning and PtA activity in a more precise manner we used TRAP to image calcium activity specifically in neurons that expressed c-Fos during fear conditioning. The next sections describe the Fos-Cre-GCaMP transgenic mice used for this purpose as a proof-of-principle to capture and image calcium transients in learning-activated PtA neurons.

### 2.3. Characterization of Fos-Cre-GCaMP Transgenic Mice

First, Fos^CreER^ and Ai38 (RCL-GCaMP3) transgenic mice were crossed to obtain a double transgenic line Fos-Cre-GCaMP. Next, we performed the genotyping of Fos-Cre-GCaMP offspring mice. As was expected, we found specific sites for GCaMP (226 bp) and Fos-Cre (293 bp) compared to 128 bp and 215 bp from DNA of wild-type mice.

**Figure 5:**
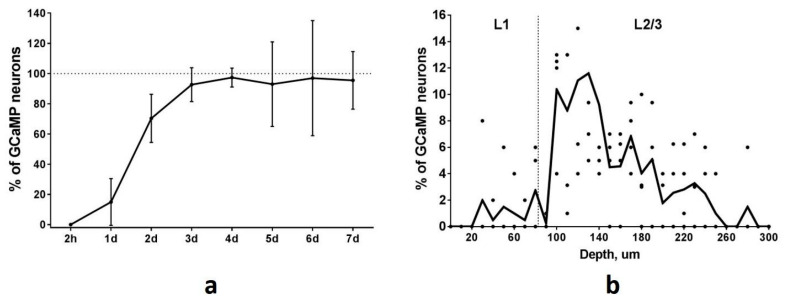
(**a**) GCaMP accumulation in the PtA neurons of Fos-Cre-GCaMP transgenic mice after cued fear learning. The total number of the GCaMP-positive neurons reached a maximum on the 4th day after training and remained at maximum level up to the 7th day (*n* = 4, mean, 95% CI). (**b**) Depth distribution of the GCaMP-expressing neurons in the PtA. Dots show the individual values for each mouse.

To determine the time course of GCaMP accumulation in TRAPed neurons we performed repeated two-photon imaging of the PtA area at different time points after the session of FC-triggered Cre-recombination. The first visualization was performed two hours after FC training to investigate the possibility of early spontaneous recombination. We found no GCaMP-expressing neurons in PtA at this time point. Thus, no background or spontaneous recombination events appear in a short time after tamoxifen injection. One day after FC training the number of GCaMP-expressing neurons was at a 15% level of the maximal number of all identified neurons (Figure 5a). We found 70% GCaMP-expressing neurons two days after the recombination event. The total number of Fos-TRAPed neurons reached 15, 35, 82, and 134 for 4 mice by the 4th day after the recombination event and remained at the same level during the following imaging sessions (Figure 5a). Thus, the maximum level of GCaMP accumulation occurs on the 4th day after the Cre-recombination and persists thereafter. Based on this data, we started calcium imaging sessions in the Fos-TRAPed neurons from the 4th day after the FC-induced recombination.

### 2.4. Two-Photon Imaging of TRAPed Neurons in the PtA

**Figure 6:**
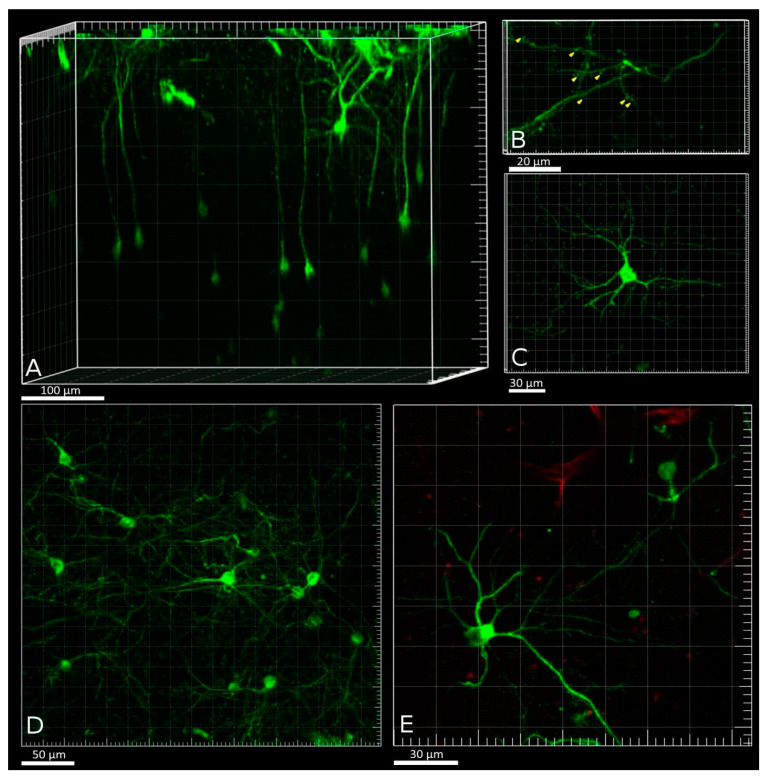
(**A**) 3D reconstruction of GCaMP-expressing neurons in the PtA. (**B**) Dendritic shafts and spines (tagging by yellow arrows) at cortical layer 1 of the PtA. (**C**–**E**) Examples of GCaMP-positive neurons in cortical layer 2 of the PtA. Green channel: GCaMP signal, red channel: autofluorescence of collagen at the brain surface.

Volumetric two-photon reconstruction of a field of view (FOV) within the PtA was performed before calcium activity imaging in five Fos-Cre-GCaMP mice. The number of detected Fos-TRAPed neurons varied in the examined animals: 20, 30, 60, 70, and 86 neurons per mouse in 0.08 mm^3^ volume. Neurons were visible at depths up to 450 μm. The maximum density of Fos-TRAPed GCaMP-expressing neurons was detected at 100–180 μm from the brain surface, a depth that matches the position of layer 2/3 of the PtA cortex (Figure 5b). Fos-TRAPed neurons showed fluorescence in the neuronal soma (nearly 15 μm in diameter), as well as in the processes (Figure 6A,B). Also, we visualized dendritic shafts (1–2 μm in diameter) with spines at depth of 5–20 μm under the brain surface (Figure 6B). Most of the Fos-TRAPed cells were pyramidal neurons according to the observed morphology (Figure 6C–E). This result is consistent with our data on the types of FC-induced Fos-TRAPed neurons in the mouse neocortex [30].

**Figure 7:**
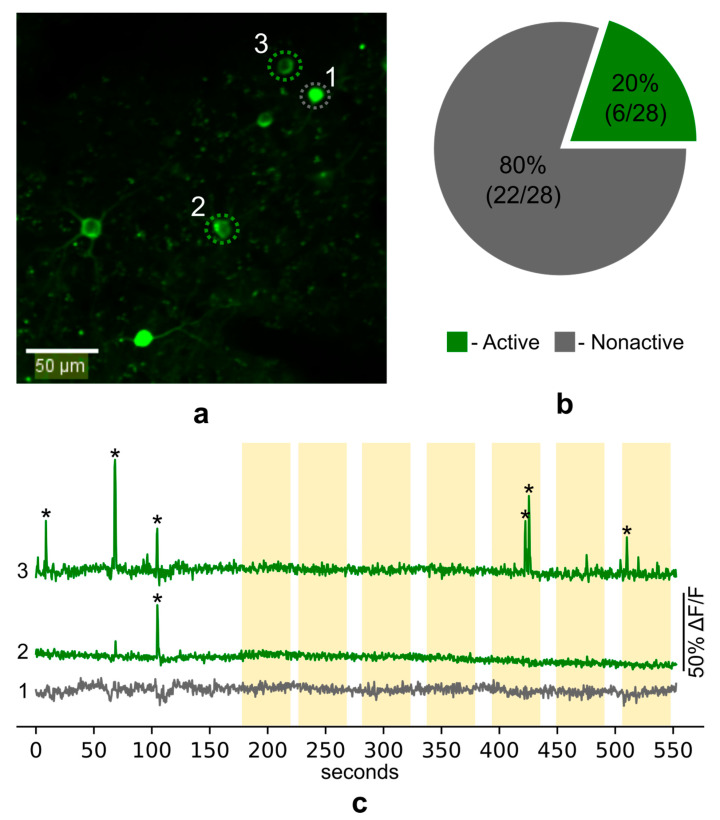
(**a**) Example field of view during in vivo imaging of Fos-Cre-GCaMP neurons; green-colored ROI—example of active cells, grey-colored ROI—example of nonactive cells. (**b**) Percentage of active and silent neurons (*n* = 3 mice). (**c**) Example calcium traces of Fos-TRAPed PtA neurons during presentation of the auditory CS. Asterisks * mark calcium events.

To examine calcium activity in the Fos-TRAPed PtA neurons during fear memory recall we presented head-fixed mice with the CS and simultaneously recorded GCaMP fluorescence on the fourth day after FC training. In total, we recorded calcium activity in the somas of 28 neurons in layer 2/3 of PtA (Figure 7a). We found 11 unique calcium events in 6 neurons. Most of the neurons (80%) showed no calcium spikes during imaging sessions (Figure 7b). Surprisingly, no specific increase of calcium activity (i.e., reliably repetitive increase) during the presentation of the CS was observed (Figure 7c).

**Figure 8:**
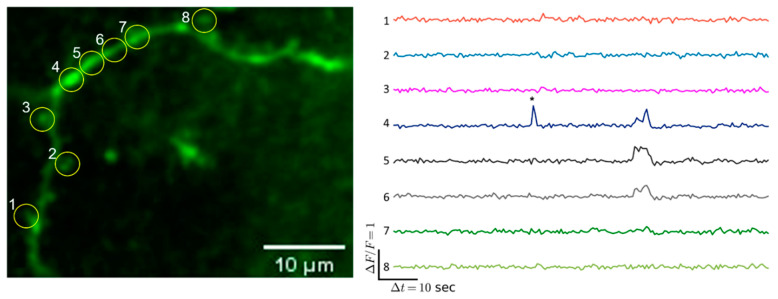
Example calcium traces in the dendritic spine of Fos-TRAPed PtA neuron. Yellow circles are regions of interest. We registered calcium event in ROI 4 and the following fluorescence increase in the neighboring ROIs 5 and 6 of the dendritic shaft. Asterisks * mark calcium events.

Additionally, spontaneous calcium transients were registered in the dendritic shaft and spines of the Fos-TRAPed neurons. Figure 8 shows an increase of GCaMP fluorescence in one area of the shaft and the following increase of fluorescence in the neighboring areas of the dendrite.

Taken together, these results suggest that the Fos-Cre-GCaMP mice are suitable for the investigation of calcium activity in the neurons, which were specifically activated during a particular learning episode.

## 3. Discussion

Although it is known that the rodent parietal cortex is involved in various forms of sensory processing and decision-making tasks, it is less clear how this area contributes to associative memory encoding and retrieval [3,4]. Our results suggest that the anterior part of the parietal cortex (the parietal association cortex) is specifically activated during episodes of the cued associative fear memory encoding and retrieval. This conclusion is supported both by immunohistochemical analysis of c-Fos expression and by in-vivo activity imaging in Fos-EGFP mice.

A possible caveat in this conclusion appears whenever it is not possible to differentiate between sensory-induced and experience-dependent processes. According to previous studies, the parietal cortex participates in multisensory processing and receives diverse projections from other sensory brain regions [3,4]. Therefore, the increased number of c-Fos-expressing cells in PtA after the presentation of the auditory stimulus during FC training or fear memory retrieval may potentially reflect this sensory processing function of the parietal cortex. To exclude this explanation, we compared changes of the PtA activity in the Paired group with the CS-only control group, which received the same set of auditory stimuli without the subsequent foot shock exposure. Using such comparison in c-Fos-immunohistochemistry experiment, we found that PtA neurons were specifically activated in the Paired group during memory retrieval. In vivo Fos-EGFP imaging showed, in addition, the specific changes of activity during both FC training and memory retrieval. These results are consistent with the hypothesis that PtA is specifically involved in encoding and retrieval of associative memory. The diverging results of the two imaging approaches can be explained by different methods used for estimation of cell populations: by ex vivo c-Fos immunohistochemical analysis we compared the whole populations of activated neurons, while using in vivo analysis we compared cells, which were inactive before and changed their activity specifically during FC or memory test. Noticeably, in Fos-EGFP mice, we showed that the predominant proportion of identified neurons expressed Fos-EGFP during all two-photon imaging sessions. This observation is consistent with previous reports on Fos-EGFP expression in the mouse cerebral cortex [18,30,31].

Taken together our ex vivo and in vivo Fos imaging data suggest that the PtA neurons are actively engaged in fear conditioning processes. This highlights a potential role of PtA in associative memory functions as well as in fear- and anxiety-related psychiatric disorders.

In addition to Fos activity imaging, we used Fos-associated transgenic mouse approach as a proof-of-principle for long-term investigation of calcium activity in neurons specifically tagged during a cognitive episode. Currently, different IEG-based methods such as compartment analysis of temporal activity by fluorescent in-situ hybridization (catFISH) or TetTag and TRAP transgenic systems are used to label histochemically neurons that were activated during two behavioral episodes [25,32,33,35,36]. However, the catFISH technique has a limited tagging window that allows comparing populations of neurons that were activated in two episodes with only about 30 min time window [36]. Also, catFISH is not suitable for the investigation of the dynamic activity of labeled cells. Other approaches like TetTag and TRAP histochemical strategies allow comparing neuronal populations which were activated in two episodes spaced for at least 72 h [32,33]. In our study, we used the TRAP strategy to introduce genetically encoded calcium indicator GCaMP3 into neurons, that expressed *c-fos* during FC training. We showed gradual accumulation of GCaMP3 in the PtA neurons of Fos-Cre-GCaMP transgenic mice peaking on the 4th day after fear learning. We also detected calcium transients in such Fos-Cre-GCaMP cells and localized them both to cell bodies and dendrites of the TRAPed neurons.

However, to our surprise, we could not detect reliable calcium responses to the auditory CS. At least two potential reasons can account for such a result. One possibility relates to the laminar heterogeneity of neocortex involvement in memory storage [24,37]. In our experiments we used memory retrieval test 24 h after training, a period which qualifies as a recent memory [24,25,36]. Notably, consolidation from the recent to remote long-term memory was shown to be accompanied by a laminar reorganization of neuronal activity in the mouse parietal cortex [24]. This shift in the pattern of neuronal activation occurred from deep cortical layers at earlier times of memory storage to superficial cortical layers at later times. Due to limitations of two-photon imaging, here, we were restricted to sampling only superficial layers of the PtA. Neurons from these layers might be less involved in the recent memory retrieval compared to deeper layer cortical neurons as was previously shown for other cortical areas [24,36,37]. This hypothesis can be tested experimentally by using GRIN(Gradient-Index)-lens two-photon imaging, three-photon imaging, or miniscopes to examine the laminar distribution of Fos-Cre-GCaMP neurons responsive to CS presentation. A second possibility is that responses of all PtA engram neurons to CS mature during systems consolidation of memory, therefore these neurons would not yet be involved in CS encoding 1 day after training. This hypothesis is supported by the recent finding that 7d- and 14d-TRAPed neurons of PrL were significantly more likely to be reactivated during remote memory retrieval when compared to 1d testing [38]. Whether the PtA neurons have a response maturation profile similar to PrL is an important question that requires a specific study.

Altogether our results indicate the impication of the parietal association cortex in associative fear learning and emphasize the potential role of PtA in fear- and anxiety-related psychiatric disorders.

## 4. Materials and Methods

### 4.1. Animals

Male C57Bl/6J mice (2–3 months old) were used for ex vivo study of *c-fos* expression in the PtA after cued fear conditioning or memory retrieval. Transgenic Fos-EGFP male and female mice (B6.Cg-Tg(Fos/EGFP)1–3Brth/J, JAX Stock No: 014135, The Jackson Laboratory) were used for in vivo two-photon imaging of PtA neurons during FC or memory retrieval. Transgenic Fos-Cre-GCaMP male and female mice were obtained by crossing two transgenic mouse lines Ai38 (RCL-GCaMP3) (B6;129S-*Gt(ROSA)26Sor^tm38(CAG-GCaMP3)Hze^*/J, JAX Stock No: 014538, The Jackson Laboratory) and Fos^CreER^ (B6.129(Cg)-*Fos^tm1.1(cre/ERT2)Luo^*/J, JAX Stock No: 021882, The Jackson Laboratory).

Wild-type mice were group-housed 5–6 per cage. Transgenic mice were housed individually. All animals were kept under a 12 h light/dark cycle. All experiments were performed during the light phase of the cycle. All methods for animal care and all experiments were approved by the National Research Center “Kurchatov Institute” Committee on Animal Care (protocol code NG-1/109PR, date of approval 13 February 2020) and were in accordance with the Russian Federation Order Requirements N 267 M3 and the National Institutes of Health Guide for the Care and Use of Laboratory Animals.

### 4.2. Behavior

During cued fear conditioning mice were placed into the fear conditioning chamber (MED Associates Inc.) for a 3-min exploration of context A. Then seven conditioned sound stimuli followed by a foot shock (2 s, 0.75 mA for wild type mice or 1 mA for the transgenic mice) were presented with ITIs 40–60 s. Each CS consisted of 5 presentations of a tone (2 s, 9 kHz, 80 dB) with 2 s intervals. 24 h later cue memory was tested in context B. Mice were placed in context B for 3-min exploration, and then presented with the CS for 3 min (45 tone signals with 2 s intervals). Context A and B were cleaned before and after each session with 70% ethanol or 53% ethanol solution of peppermint, respectively. Context A was an IR light illuminated plastic box (30 cm × 23 cm × 21 cm) with a grid floor. To change the context for memory retrieval we placed the black plastic A-shaped insert into the FC chamber and covered the grid floor with a plastic sheet and wood sawdust on top (context B). Context B was illuminated with white and IR light. Freezing behavior was quantified using an automatic detection system (Video Freeze, MED Associates Inc., Fairfax, VT, USA).

For c-Fos immunostaining three groups of mice were used: Paired (*n*(FC) = 12, *n*(test) = 11), CS-only(*n*(FC) = 10, *n*(test) = 11) and Home cage group (*n*(FC) = 12, *n*(test) = 13). Mice from the Paired group were conditioned using the protocol described above. Mice from the CS-only group were submitted to the same protocol but without foot shock. Half of the mice were sacrificed 90 min after FC (*n*(FC)), while the other half was tested for memory retrieval and sacrificed 90 min after a test (*n*(test)). The Home cage mice were sacrificed in parallel with experimental animals without any behavioral manipulations.

Fos-EGFP mice were divided into the same groups: Paired (*n* = 7), CS-only (*n* = 6) and Home cage (*n* = 3). One month after cranial window implantation mice undergo FC training and memory retention test as described above.

Fos-Cre-GCaMP mice were FC trained 24 h after TM injection according to the described protocol.

### 4.3. Genotyping

Fos-Cre-GCaMP mice were genotyped at the age of 30–60 days. For genotyping we extracted DNA from the tail tissue in lysis buffer (0.01 M Tris-HCl, pH = 7.5; 0.01 M EDTA, pH = 8.0; 0.1 M NaCl; 1% SDS; 0.2 mg/mL proteinase K), performed PCR (in PCR buffer with Taq polymerase, DNTP, forward and reverse primers) and identified DNA sites using electrophoresis in agarose gel (2.5%). Primers oIMR4981, oIMR8038, 34,319, 34,962 were used for GCaMP genotyping, and 17,016–17,018 for Fos-Cre genotyping (The Jackson Laboratory).

### 4.4. Surgery

For cranial window surgery, transgenic mice were anesthetized with an intraperitoneal injection of zoletil (0.04 mg/g body weight) and xylazine (0.5 µg/g body weight). Dexamethasone (4 mg/kg) was administered subcutaneously 5 min before surgery to prevent tissue stress and cerebral edema. Viscotears moisturizing gel (Novartis Healthcare) was applied to prevent eye drying. Mice were fixed in a stereotaxic frame (Stoelting) and 37 °C body temperature was maintained by a heating plate (Physitemp). 3 mm craniotomy over the PtA (centered 1.0 mm lateral and 1.7 mm posterior to the Bregma) [3] was performed as described previously [39]. A 5-mm round glass coverslip (Menzel, Thermo Fisher) was attached to the skull using cyanoacrylate glass glue (Henkel). A Neurotar head post (Neurotar Ltd., Helsinki, Finland) was cemented to the skull with dental cement (Stoelting) and was later used for head fixation in the Mobile Home Cage system (MHC, Neurotar Ltd., Helsinki, Finland).

Two weeks after surgery Fos-EGFP and Fos-Cre-GCaMP mice were head-fixed in the MHC each day (5 to 40 min) for two weeks for habituation to imaging conditions. In this system, a head-fixed mouse can move around a lifted MHC and freely explore its environment [40].

### 4.5. Tamoxifen Injection

Fos-Cre-GCaMP mice received a single i.p. injection (150 mg/kg) of TM (Sigma) 24 h before FC training. TM was dissolved in the corn oil (Sigma) (10 mL/kg) and 96% ethanol (1.3 mL/kg) at 65 °C for 1–2 h. The dose of TM and timing of injection were in accordance with the previously described protocol [32].

### 4.6. Immunohistochemistry

Mice were sacrificed 90 min after FC training or test. Brains were removed and immediately frozen in liquid nitrogen vapor. 20-µm coronal sections were prepared on a cryostat (Leica) and fixed in 4% paraformaldehyde. For c-Fos immunostaining, primary rabbit polyclonal antibodies against the c-Fos protein (sc-52, Santa Cruz Biotechnology, Dallas, TX, USA, dilution 1:500) and horse secondary antibodies against rabbit conjugated with avidin-biotin complex (ImPRESS reagent kit anti-rabbit, Vector Laboratories) were used. Sections were stained in 0.06% diaminobenzidine solution (Sigma). Brain sections were taken at a distance of −1.7 mm from the bregma. Whole section images were acquired through the fluorescence microscope scanner (Olympus, VS110).

### 4.7. In Vivo Two-Photon Imaging

Two-photon imaging was performed on Fos-EGFP and Fos-Cre-GCaMP mice 30–60 days after cranial window implantation using an Olympus MPE1000 two-photon microscope equipped with a Mai Tai Ti:Sapphire femtosecond-pulse laser (Spectra-Physics) and a water-immersion objective lens, 20 × 1.05 NA (Olympus). 960 nm wavelength was used for excitation. Series of images were recorded with the Olympus Fluoview Software Version 3.1.

#### 4.7.1. Fos-EGFP Mice

The volume series of images (0–350 μm under the pia) were recorded at 0.82 frame per second (fps) continuously, with 500 × 500 μm field of view and 512 × 512 pixels resolution. PtA two-photon imaging was performed three days before FC (basal level of c-Fos expression in the home cage), 90 min after FC, and 90 min after memory test. Home cage mice were imaged together with other groups but without any prior experience.

#### 4.7.2. Fos-Cre-GCaMP Mice

To determine the dynamics of GCaMP accumulation after Cre recombination, we visualized the PtA at different time points starting 2 h after FC in 4 mice. Volume series of images (0–300 μm under the pia) were recorded at 0.2 fps continuously, with 500 × 500 μm field of view and 512 × 512 pixels resolution.

To analyze the activity of trapped cells PtA layer 2/3 neurons (approximately 100–200 μm deep from pia) were imaged three days after FC. During the calcium imaging, mice received seven series of the CS (one series consisted of 10 short tones (2 s, 9 kHz, 80 dB) with 2 s intervals). Time series of images were recorded at 1.78 fps continuously, with 253 × 253 μm field of view, 256 × 256 pixels resolution and 2× zoom.

For spontaneous calcium activity in dendrites, we recorded a time series of images at 1.92 fps, with 35 × 44 μm field of view, 180 × 144 pixels resolution and zoom 4× at the depth of 5–10 μm without any stimulation.

The image analysis was performed in Olympus Fluoview Software Version 3.1, FIJI, Imaris 7.4.2 and a custom Python plugin. Regions of interest (ROIs) corresponding to identifiable cell bodies or spines were selected manually in the Olympus Fluoview Software Version 3.1. ΔF/F was calculated by subtracting each value with the mean of the lower 50% of previous 10-s values and dividing it by the mean of the lower 50% of previous 10-s values.Calcium events detection was performed whenever the difference between a trace amplitude and its median value crossed the threshold of 4 median absolute deviations, calculated for each cell over the whole trace.

## Data Availability

Data is contained within the article.
